# Associations between adverse childhood experiences and perinatal outcomes

**DOI:** 10.1016/j.jped.2025.101433

**Published:** 2025-10-03

**Authors:** Nina de Siqueira Kuperman, Maria Clara Magalhães-Barbosa, Fernanda de Carvalho Lima, Mariana Barros Genuino de Oliveira, Jaqueline Rodrigues Robaina, Margarida dos Santos Salú, Arnaldo Prata-Barbosa, Antônio José Ledo Alves da Cunha

**Affiliations:** a Universidade Federal do Rio de Janeiro (UFRJ), Faculdade de Medicina, Programa de Pós-Graduação em Medicina Interna, Rio de Janeiro, RJ, Brazil; b Instituto D'Or de Pesquisa e Ensino (IDOR), Departamento de Pediatria, Rio de Janeiro, RJ, Brazil; c Universidade Federal do Rio de Janeiro (UFRJ), Faculdade de Medicina, Departamento de Pediatria, Rio de Janeiro, RJ, Brazil

**Keywords:** ACE-IQ, Adverse experiences in childhood, Child abuse and neglect, Gestational outcomes

## Abstract

**Objective:**

To determine the frequency of adverse childhood experiences (ACE) among a cohort of pregnant women (primary outcome) and explore their association with prematurity, pre-eclampsia, and fetal growth restriction (secondary outcomes).

**Methods:**

The Adverse Childhood Experiences International Questionnaire - ACE-IQ was applied to patients during prenatal visits. Information on perinatal outcomes was collected from medical records. The proportion of total ACE and its different domains was estimated. Multiple logistic regressions were performed to assess the association between ACE and outcomes, after adjusting for possible confounding factors.

**Results:**

A cohort of 307 pregnant women completed the ACE-IQ. The results in the binary and frequency versions were, respectively: mean (SD) scores of 5.84 (2.87) and 3.55 (2.73); the proportion of ACE 4 of 75% and 44%; the most prevalent ACE domains were home dysfunction (89.6 and 86.6%) and exposure to community violence (76.5 and 50%). For fetal growth restriction, pregnant women with ACE ≥ 4 had 2.32 times (95% CI: 1.04–5.37; p = 0.042) higher chance of this outcome. For preterm birth, the odds ratio was 1.1 (95% CI: 0.5–2.6; p = 0.886), indicating no statistically significant association. There was no significant association between the total ACE score, or its domains, and the other perinatal outcomes studied.

**Conclusions:**

The frequency of ACE was high in this cohort of pregnant women, and exposure to community violence was associated with fetal growth restriction. The investigation of the association with other perinatal outcomes should be extended to a general population of pregnant women.

## Introduction

According to the Centers for Disease Control and Prevention (CDC), adverse childhood experiences (ACEs) are potentially traumatic events that occur in childhood (0–17 years), including abuse, neglect, and household dysfunction that can undermine a child’s sense of safety, stability, and bonding [1].

Increasing evidence suggests that adverse consequences of childhood maltreatment may be associated with various physical and mental health outcomes in adulthood, such as chest pain, hypertension, some cancers, obesity, acute myocardial infarction, depression, anxiety, substance abuse, and schizophrenia [2, 3]. This effect seems to be not only restricted to exposed individuals but can also be epigenetically transmitted to their children [4].

At least one in five women in the world has a history of maltreatment during childhood [5]. Adverse experiences that occurred before the age of 18 in a woman’s life seem to have a lasting impact on reproductive health, with untoward effects on the fetus by increasing the risk of prematurity and low birth weight [3, 6, 7]. In addition, a dose-response association has been observed between the number of ACE and the risk for excessive alcohol consumption, drug use, and smoking in gestation[8] as well as a greater propensity to depression and anxiety [3,7]. The mother-infant bond can be compromised by the experience of neglect in maternal infancy, and mothers who have experienced ACE are also more likely to neglect the care of their children [9]. In addition, vertical transmission of the Human Immunodeficiency Virus (HIV) is increased in women in these situations [10]. Toxic responses to stress, including activation of the hypothalamic-pituitary-adrenal axis and inflammation, are the main mechanisms hypothesized to explain how ACEs can cause biological changes that impact health outcomes [8].

The World Health Organization (WHO) encourages researchers to assess the occurrence of ACE and its association with adverse outcomes in adulthood [5]. The frequency of ACE in pregnant women and the possible association with adverse perinatal outcomes are still poorly reported in the current literature. This study aims to describe the frequency of ACE in a cohort of pregnant women and to explore the association between adverse experiences in maternal childhood and perinatal outcomes such as prematurity, fetal growth restriction, and preeclampsia [4, 8, 9].

Despite growing international evidence, studies assessing the prevalence of Adverse Childhood Experiences (ACEs) and their associations with perinatal outcomes remain scarce in low- and middle-income countries (LMICs), especially in Brazil. High-risk pregnant women in Brazil are more likely to be exposed to structural vulnerabilities that may both increase their likelihood of experiencing ACEs and exacerbate their effects on maternal and fetal health. However, few studies have addressed this issue in public referral hospitals, limiting the understanding of the intergenerational consequences of ACEs in socially vulnerable populations [11,12].

## Methods

### Study design and participants

A prospective observational study in a cohort of pregnant women was conducted to assess the frequency of ACE and the association between ACE and perinatal outcomes.

A convenience sample was used, composed of consecutive pregnant women 18 years of age or over in the first appointment at the high-risk prenatal clinic of the Maternity School of the Federal University of Rio de Janeiro between November 2018 and August 2019. During this period, approximately 1200 deliveries occurred, and 343 women were invited to participate. Of these, 307 met the inclusion criteria and were enrolled. The decision to adopt a convenience sample was based on feasibility, staff availability, clinic flow, and ethical concerns.

About 80 % of the patients attending are pregnant women with diabetes, hypertension, cardiopathies, fetal malformations, or other risk conditions, referred to prenatal care from the beginning of pregnancy. Other patients are previously normal pregnancies referred to basic health units, where they begin prenatal care, and are referred already with advanced gestational age to the reception at ME-UFRJ, where they will be given continuity of prenatal care and childbirth.

### Data collection, measurements, and procedures

Sociodemographic data such as age, marital status, race/ethnicity, educational level, and reports of adverse experiences during childhood were retrieved from a self-reported questionnaire applied to the participants during the first prenatal visits, the Adverse Childhood Experiences International Questionnaire (ACE-IQ). Data on prenatal care and childbirth, including variables on risk behaviors (alcohol intake and smoking) and gestational outcomes, such as preeclampsia (PE), fetal growth restriction (FGR), and prematurity (PMT), were retrieved from medical records.

The authors used directed acyclic graphs (DAGs) to identify minimal sufficient adjustment sets for each outcome. The adjustment set included maternal age, parity, education, and smoking during pregnancy.

### Adverse childhood experiences international questionnaire - ACE-IQ

The ACE-IQ was translated and cross-cultural adapted to Brazilian Portuguese, according to international protocols. It is the standard instrument proposed by the WHO for use in different countries of the world, allowing comparison between studies in different populations. The ACE-IQ is to be applied to adults aged 18 years or older to assess the occurrence of adverse experiences in the past during their childhood. In addition to items on sociodemographic data, the ACE-IQ has 32 items grouped into 13 domains: emotional abuse; sexual abuse; alcohol and/or drug abuse at home; incarcerated household member; member with chronic depression, mental health problem or suicidal ideation; family member treated violently; parental separation or divorce; emotional neglect; physical neglect; bullying; community violence; collective violence. There are two versions: a binary version with yes/no responses; and a frequent version with Likert responses (often/ sometimes/ once/ never). In the binary version, each domain receives a score = 1, if at least one of the items belonging to it receives a YES response, regardless of the number of times the abuse has occurred. In the frequency version, each domain receives a score = 1 only if at least one of the items belonging to the respective domain occurred frequently ("sometimes" or "often"). In both cases, the resulting score ranges from 0 to 13 [5,13]. The authors used the binary scoring version of the ACE-IQ, translated and adapted to Brazilian Portuguese according to ISPOR recommendations [14].

### Perinatal outcomes

Preeclampsia (PE) was defined as the presence of hypertension in pregnancy associated with urinary proteinuria greater than or equal to 300 mg in 24 h. Fetal growth restriction (FGR) was defined as estimated fetal weight at ultrasonography below the 10th percentile for gestational age or birth weight at z score < −2 (small newborn for gestational age - SGA). Prematurity (PMT) was defined as gestational age at birth <37 weeks.

### Data analysis

Categorical variables were described as proportions, and continuous variables as means and standard deviations or medians and interquartile intervals.

To evaluate the association between ACE (independent variable) and each of the three gestational outcomes (dependent variables) the following steps were adopted: 1) identification, on a theoretical basis, of the minimum set of confounding variables through Directed Acyclic Graphics (DAG), built in the DAGITY program; 2) performing multiple logistic regressions to estimate odds ratios (OR) with adjustment for the possible confounding factors identified.

The analysis was conducted using R software, version 4.1.1. Three outcomes were considered: prematurity, preeclampsia, and FGR/SGA, with ACE-IQ as the exposure variable. Initially, a bivariate analysis was performed to assess the relationship between selected variables (ACE-IQ, education level, skin color) and the outcomes, using the chi-square statistic. Following this, an adjusted regression model was applied to further examine the associations.

In this study, the authors evaluated the frequency of ACE and its association with perinatal outcomes using both binary and frequency versions. The authors used the ACE-IQ score as a variable categorized in two ways: 1, 2, 3, and 4, or <4, 4–6, and 7. The authors estimated, separately, the frequency of nine of the 13 ACE domains, gathered similarly as previously performed in the literature^21^ in intrafamilial ACE (physical abuse, emotional abuse, sexual abuse, domestic dysfunction, emotional neglect, physical neglect) and social ACE (peer violence, the testimony of community violence, exposure to war/collective violence). The authors conducted a sensitivity analysis comparing included participants (*n* = 307) and those lost to follow-up (*n* = 36). The comparison included maternal age, education, income, employment, parity, abortion history, and tobacco use. No significant differences were found, indicating minimal attrition bias (see Supplementary Table 1).

Data from all included participants were used for the frequency study. For the association analysis, data were used only from women who had childbirth in the institution, excluding those who had abortions or childbirth in another institution.

### Ethical issues

The study was approved by the Research Ethics Committee of ME-UFRJ. All patients received information about the study and were assured that participation was optional, not impacting the follow-up in the unit. Patients were included if they read and signed an informed consent form (ICF).

## Results

All 307 participants addressed during the period responded to the ACE-IQ. The mean age was 30.57 years (SD = 6.79), 53.7 % were brown, 55.4 % had completed high school, and 72.9 % lived with a partner (Table 1).

**Table 1:** Sociodemographic and clinical characteristics of pregnant women during prenatal visits in the Maternity Hospital of the Federal University of Rio de Janeiro (ME-UFRJ). from November 2018 to August 2019.

Characteristics	*n* = 307
Age - mean (± SD)	30.57 (± 6.79)
Race/ethnicity- n ( %)	
White	78 (25.4 %)
Black	56 (18.2 %)
Brown	165 (53.7 %)
Asian	3 (1.0 %)
Native	3 (1.0 %)
Refused to answer	2 (0.7 %)
Educational Status - n ( %)	
No formal schooling	3 (1.0 %)
Less than primary school	56 (18.2 %)
Primary school completed	29 (9.4 %)
Secondary/High school completed	170 (55.4 %)
College/High School completed	32 (10.4 %)
Postgraduate degree	17 (5.5 %)
Work Status - n ( %)	
Government employee	17 (5.5 %)
Formal non-government employee	114 (37.1 %)
Self-employed	74 (24.1 %)
Non-paid	1 (0.3 %)
Student	11 (3.5 %)
Homemaker	49 (16.0 %)
Retired	0
Unemployed (able to work)	39 (12.7 %)
Unemployed (unable to work)	1 (0.3 %)
Refused to answer	1 (0.3 %)
Marital Status - n ( %)	
Married	99 (32.2 %)
Living as a couple	125 (40.7 %)
Divorced or separated	9 (2.9 %)
Single	72 (23.5 %)
Widowed	1 (0.3 %)
Other	1 (0.3 %)
Gestational Outcomes	*n* = 266
Pre-eclampsia	34 (12.8)
Fetal Growth Restriction	29 (10.9)
Prematurity	38 (14.3)

The 36 participants lost to follow-up did not differ significantly from those included in the analysis across key sociodemographic and obstetric variables (*p* > 0.05 for all comparisons).

Of this total, 40 patients (13 %) were lost, since four of them have suffered a miscarriage, and 36 have given birth in another institution, making it impossible to obtain information about the gestational outcomes. Of the 266 pregnant women who had the study outcomes available in the medical records, 34 (12.7 %) had pre-eclampsia (PE), 29 (10.9 %) had fetal growth restriction (FGR), and 38 (14.2 %) had prematurity (PMT). There was an overlap between FGR and PE (*n* = 1), between FGR and PMT (*n* = 5), between PE and PMT (*n* = 10), and between the three outcomes (*n* = 6) (Table 1).

In the binary version, the mean ACE-IQ score was 5.84 (2.87). The frequency of ACE ≥ 4 was 75 %, 33.5 % from 4 to 6, and 41.4 % ≥ 7. In the frequency version, the average score was 3.55 (2.73), and 44 % of pregnant women had ACE ≥ 4, 30 % from 4 to 6, and 14 % ≥ 7. The most frequent domain in the category of intrafamilial ACE was home dysfunction, with a frequency of 89.6 % in the binary version and 86.6 % in the frequency version, followed by emotional (66,8 % and 64,2 %, respectively) and physical abuse (58 % and 53,7 %, respectively). In the category of social ACE, the most frequent domain was to witness violence in the community, with a frequency of 76.5 % in the binary version and 56 % in the frequency version (Table 2).

**Table 2:** Mean score and frequency of Adverse Experiences in childhood (ACE) in pregnant women (*n* = 307) according to the type of ACE-IQ score: binary and frequency.

ACE-IQ score	ACE-IQ score version			
	Binary		Frequency	
mean (± SD)	5.84 (± 2.87)		3.55 (± 2.73)	
	n	% (IC 95 %)	n	% (IC 95 %)
0	6	2.0 (0.9; 4.2)	27	8.8 (6.1; 12.5)
1	14	4.6 (2.7; 7.5)	57	18.6 (14.6; 23.3)
2	21	6.8 (4.5;10.2)	49	16.0 (12.3; 20.5)
3	36	11.7 (8.6; 15.8)	39	12.7 (9.4; 16.9)
4+	230	74.9 (69.8; 79.4)	135	44 (38.5; 49.6)
1–3	77	25.1 20.6; 30.2)	172	56 (50.4; 61.5)
4–6	103	33.5 (28.5; 39.0)	91	29.6 (24.8; 35.0)
7+	127	41.4 (36.0; 47.0)	44	14.3 (10.9; 18.7)
Intrafamilial ACE				
Physical neglect	135	44 (38.5; 49.6)	127	41.4 (36.0; 47.0)
Emotional neglect	90	29.3 (24.5; 34.6)	90	29.3 (24.5; 34.6)
Emotional abuse	205	66.8 (61.3; 71.8)	178	58 (52.4; 63.4)
Physical abuse	197	64.2 (58.7; 69.3)	165	53.7 (48.2; 59.2)
Sexual abuse	108	35.2 (30.1; 40.7)	52	16.9 (13.2; 21.5)
Household dysfunction	275	89.6 (85.7; 92.5)	266	86.6 (82.4; 90.0)
Social ACE				
Peer violence	132	43.0 (37.6; 48.6)	119	38.8 (33.5; 44.3)
Community violence	235	76.5 (71.5; 80.9)	172	56.0 (50.4; 61.5)
Collective violence	86	28.0 (23.3; 33.3)	48	15.6 (12.0; 20.1)
Total n ( %)	307	100	307	100

ACE-IQ, Adverse Childhood Experiences – International Questionnaire.

Directed acyclic graphs identified race and educational level as the minimum set of variables to adjust for the associations between ACE and three outcomes studied (supplementary Table 1). There was no significant association between adverse experiences in childhood and the outcomes of prematurity and preeclampsia, either with the binary or frequency versions of the ACE-IQ, both categorized in two levels (score < 4 or ≥ 4) or three levels (score < 4, 4 to 6 or ≥ 7). For fetal growth restriction, pregnant women with ACE ≥ 4 had a 2.32 times higher chance of this outcome compared to pregnant women with ACE < 4. This association was only present with ACE from 4 to 6 but did not occur with ACE ≥ 7 (*p* = 0.247). No significant difference was observed between the coefficients of the two strata - ACE from 4 to 6 and ACE ≥ 7 (*p* = 0.683) (Table 3).

**Table 3:** Association between adverse childhood experiences and perinatal outcomes in pregnant women (*n* = 266).

		PMT^a^			PE^a^			FGR^a^		
ACE-IQ	N	OR (CI 95 %)	p-value		OR (CI 95 %)	p-value		OR (CI 95 %)	p-value	
**BINARY (2 levels)**										
< 4 (ref.)	64	-	-		-			-		
≥ 4	200	1.1 (0.5; 2.6)	0.886		0.6 (0.3; 1.4)	0.204		2.3 (0.8; 7.9)	*0.152*	
**BINARY (3 levels)**										
< 4 (ref.)	64	-			-			-		
4–6	92	1.0 (0.4; 2.6)	0.954		0.81 (0.3; 2.0)	0.651		2.2 (0.7; 8.3)	*0.201*	
≥ 7	108	1.1 (0.5; 2.9)	0.779		0.4 (0.2; 1.1)	0.082		2.3 (0.8; 8.4)	*0.166*	
**FREQUENCY (2 levels)**										
< 4 (ref.)	149	-			-			-		
≥ 4	115	1.2 (0.6; 2.4)	0.676		0.6 (0.3; 1.3)	0.182		2.3 (1.0; 5.4)	***0.042***^c^	
**FREQUENCY (3 levels)**										
< 4 (ref.)	149	-			-					
4–6	76	1.3 (0.6; 2.9)	0.514		0.8 (0.3; 1.7)	0.522		2.5 (1.0; 6.0)	*0.041*^b^*^,^*^c^	
≥ 7	39	0.9 (0.3; 2.5)	0.869		0.3 (0.0; 1.1)	0.103		2.0 (0.6; 6.0)	*0.247*^b^	

ACE-IQ, Adverse Childhood Experiences – International Questionnaire; PE, pre-eclampsia; OR, *odds ratio*; PMT, prematurity; FGR, fetal growth restriction.

a Adjustment for age and race/ethnicity in the models of the three outcomes (PMT. PE and FGR).

b No significant difference between the coefficients of the two strata (score 4–6 and score ≥ 7) was observed (*p* = 0.683).

c *p* < 0.05.

The association between each domain of the ACE-IQ and the outcomes was significant for exposure to community violence and fetal growth restriction (*p* < 0.05) and borderline for the association of sexual abuse and prematurity (*p* < 0.10) and the association of emotional neglect and fetal growth retardation (*p* < 0.10) (Table 4).

**Table 4:** Association between the domains of adverse childhood experiences and perinatal outcomes in pregnant women (*n* = 266): prematurity, pre-eclampsia, and fetal growth restriction.

		PMT^a^		PE^a^		FGR^a^	
ACE	N	OR (CI 95 %)	p-value	OR (CI 95 %)	p-value	OR (CI 95 %)	p-value
Intrafamilial	253	0.6 (0.1; 4.2)	*0.550*	0.5 (0.1; 3.6)	*0.423*	0.5 (0.1; 3.5)	*0.416*
Physical neglect	118	1.2 (0.6; 2.5)	*0.609*	1.3 (0.6; 2.7)	*0.541*	0.8 (0.3; 1.8)	*0.566*
Emotional Neglect	42	1.7 (0.7; 3.9)	*0.232*	0.9 (0.3; 2.3)	*0.787*	2.3 (0.9; 5.7)	***0.069***^c^
Emotional abuse	175	1.0 (0.5; 2.1)	*0.976*	0.6 (0.3; 1.4)	*0.255*	2.1 (0.9; 6.0)	*0.121*
Physical abuse	168	1.2 (0.6; 2.5)	*0.677*	0.5 (0.3; 1.2)	*0.115*	2.0 (0.8; 5.2)	*0.142*
Sexual abuse	95	2.0 (1.0; 4.0)	***0.06***^c^	0.5 (0.2; 1.2)	*0.121*	0.9 (0.4; 1.9)	*0.698*
Household dysfunction	236	1.4 (0.5; 6.3)	*0.599*	0.6 (0.2; 1.9)	*0.328*	1.6 (0.4; 10.1)	*0.565*
Social	225	0.8 (0.3; 2.2)	*0.662*	0.5 (0.2; 1.4)	*0.175*	2.5 (0.7; 16.2)	*0.221*
Peer violence	114	1.3 (0.6; 2.7)	*0.456*	0.8 (0.3; 1.6)	*0.470*	1.1 (0.5; 2.4)	*0.879*
Community violence	206	0.7 (0.3; 1.5)	*0.319*	0.5 (0.2; 1.3)	*0.140*	4.5 (1.3; 28.9)	***0.047***^b^
Collective violence	74	0.8 (0.3; 1.6)	*0.495*	0.7 (0.3; 1.6)	*0.407*	1.3 (0.6; 2.9)	0.533

ACE-IQ, Adverse Childhood Experiences – International Questionnaire; PE, pre-eclampsia; OR, *odds ratio*; PMT, prematurity; FGR, fetal growth restriction.

a Adjustment for educational level and color according to the results of the directed acyclic graphs for each outcome.

b *p* < 0.05.

c *p* < 0.10.

## Discussion

To our knowledge, this was the first study conducted in Brazil on the prevalence of ACE in pregnant women and its association with perinatal outcomes such as preeclampsia, prematurity, and fetal growth restriction. Few studies have evaluated this association in the world literature. In this cohort of pregnant women from a high-risk maternity hospital, the frequency of ACE was high, but there was no significant association with the perinatal outcomes studied.

The prevalence reported in this study does not reflect the general population, but rather the specific cohort of high-risk pregnant women treated in a Brazilian referral hospital, which limits the external validity of the findings. Besides, the use of a convenience sample was justified by the logistical and ethical challenges of conducting randomized recruitment in a public referral hospital for high-risk pregnancies.

Sensitivity analysis indicated that losses to follow-up did not introduce measurable bias, as participants lost did not differ from those included in any key sociodemographic or clinical variable.

Analyzing the results obtained with the two versions of the ACE-IQ, it is observed that the frequency estimates with the binary version are higher, suggesting greater sensitivity and lower specificity than the frequency version. The binary version has been the most recommended and widely used [10,15]. When evaluated by this version, the present study showed a frequency of ACE in pregnant women superior to other recent studies conducted in both Brazil [10,15, 16, 17, 18] and in other low and middle-income countries [10,15,18], with diverse populations and tools.

ACE-IQ may also be a more sensitive tool than other tools used in these studies, since it explores several dimensions of abuse, often with several items for each dimension. Particularly, the use of the binary version, which requires only one of the items of each dimension with a positive response to scoring the dimension, seems to be a factor that contributes to the higher frequency of ACE demonstrated in the present study. In addition, although the ACE-IQ refers to events occurring in childhood with no apparent reason for the frequency of ACE in pregnant women to differ from the general population, it should be considered that the especially sensitive moment of pregnancy can make the patients report biased compared to what could be obtained at other times in life.

In the literature, the cutoff point for discrimination between low and high ACE scores is not uniform. Even when studies using the same instruments in similar populations are compared, the cut-off points differ widely. In pregnant women, the frequency of ACE varies according to the instrument used and the country studied. Socioeconomic and cultural disparities are likely to contribute to these variations.

A study conducted in the USA investigated the presence of childhood maltreatment of pregnant women using the Childhood Trauma Questionnaire-Short Form (CTQ-SF) and showed that 45 % of the patients had experienced two or more adverse events in childhood [19]. In England, the application of a simplified instrument in the postpartum period, focusing on intrafamilial dimensions of ACE, identified that 52.8 % of the participants reported at least 1 adverse event in childhood, and 12.7 % reported 4 or more events [20]. In Tunisia, a study applying the ACE-IQ in pregnant women identified that 88.9 % of women reported a history of exposure to at least one ACE, of which 46.7 % reported three or more ACE [21]. The results of the present study show a higher frequency, since, in the binary version of ACE-IQ, 75 % had 4 or more ACE, 41 % had 7 or more ACE, and only 2 % did not report ACE.

This study, conducted in a public institution of a middle-income country such as Brazil, with many social inequalities, showed that home dysfunction and exposure to community violence were the most prevalent types of ACE. Other authors evaluated the frequency of different dimensions of ACE, using the ACE-IQ, but in non-pregnant populations.

A recent systematic review included 64 studies using ACE-IQ in community samples, most of which were conducted in Asia and Africa. On average, 75 % of participants suffered ACE, with a mean score of mainly emotional abuse and bullying. Different geographic areas showed different frequencies in the dimensions, but most studies focused on intrafamilial dimensions of ACE.

A recent study conducted in Brazil [16] in a cohort of mother-infant dyads followed from birth used a reduced version of the ACE-IQ applied to mothers during the last visit for the 4-year-old child. The version included only the nine intrafamilial dimensions of the ACE-IQ: emotional, physical, and sexual abuse, intrafamilial violence, living with addicts, mental/suicidal patients, and with prisoners, physical negligence, and loss/divorce of parents. Using the binary version, the authors reported a history of ACE in 86.8 % of mothers. The most reported ACEs were having a family member treated violently (62 %), followed by parental separation/divorce and emotional abuse (about 50 %).

In a study conducted on pregnant women in Tunisia(23), among intrafamilial ACE, the most reported type of abuse was home dysfunction (58.3 %), followed by physical abuse and emotional abuse in 40 % and 32.2 %, respectively. Witnessing community violence was the most reported social ACE (40 %), followed by peer violence (39.3 %).

In Italy, a high-income country, emotional abuse and bullying victimization appeared as childhood adversities associated with mental health disorders in adulthood.

The prevalence of Adverse Childhood Experiences (ACEs) in low- and middle-income countries (LMICs) is a significant concern, as highlighted by studies in Tunisia [21] and Mexico [22] where structural inequities exacerbate exposure to early adversity. This aligns with findings from other LMICs, such as Honduras and South Africa [23,24], where high rates of ACEs are reported, often linked to socio-economic challenges and community violence. The impact of ACEs on fetal growth and development is also notable, with evidence suggesting that emotional neglect and community violence can have distinct biological and psychosocial effects [12,24].

In contrast, research in high-income countries often focuses on the association between ACEs and conditions like preeclampsia [25] which was not observed in the Brazilian cohort studied, possibly due to population differences or the specific clinical profiles of high-risk pregnancies in Brazil [10,11]. This discrepancy underscores the need for context-specific research to understand the varied impacts of ACEs across different socio-economic and cultural settings [26, 27, 28].

## Conclusion

The frequency of ACE was high in this cohort of pregnant women and specifically, the exposure to community violence was associated with fetal growth restriction. The predominance of high-risk pregnancies may have contributed to the absence of an association between ACE and the other perinatal outcomes, prematurity, and pre-eclampsia. The investigation should be extended to a general population of pregnant women and include other adverse outcomes such as gestational diabetes, miscarriage, and depression/anxiety disorders in pregnancy.

## Authors’ contributions

NSK, MCMB, APB and AJLAC Conceptualization.

NSK and MSS Data curation.

NSK, MCMB and FCL Formal analysis.

MBGO and JRR Project administration.

NK and MCMB Writing - original draft.

All authors contributed to the writing of the manuscript, read, and approved its final format.

MCMB, AJLAC, MBGO and APB Writing - review & editing.

All authors approved the final version of the manuscript.

## Conflicts of interest

The authors declare no conflicts of interest.

## Appendix Supplementary materials

Image, application 1

## Footnotes

Supplementary material associated with this article can be found in the online version at doi:10.1016/j.jped.2025.101433.
