# Supplementary material for: Associations between adverse childhood experiences and perinatal outcomes
Source: J Pediatr (Rio J). 2025 Oct 3;101(6):101433. doi: 10.1016/j.jped.2025.101433 (PMC12744608; doi:10.1016/j.jped.2025.101433)
Supplement: Supplementary file 1 [file mmc1.docx]

**JPED-D-24-00298_ Supplementary Material**

**Supplementary Table 1**

| **Variable** | **Included (n = 307)** | **Lost to follow-up (n = 36)** | **p-value** |
| --- | --- | --- | --- |
| Age (mean ± SD) | 27.4 ± 5.9 | 27.8 ± 6.1 | 0.71 |
| ≥ High school education (%) | 80% | 78% | 0.75 |
| Income < 2 minimum wages (%) | 58% | 60% | 0.81 |
| Currently employed (%) | 32% | 30% | 0.79 |
| Primigravida (%) | 40% | 39% | 0.90 |
| History of abortion (%) | 29% | 28% | 0.92 |
| Smoked during pregnancy (%) | 10% | 11% | 0.84 |
